# Improved Plasmonic Hot-Electron Capture in Au Nanoparticle/Polymeric Carbon Nitride by Pt Single Atoms for Broad-Spectrum Photocatalytic H_2_ Evolution

**DOI:** 10.1007/s40820-023-01098-2

**Published:** 2023-05-20

**Authors:** Manyi Gao, Fenyang Tian, Xin Zhang, Zhaoyu Chen, Weiwei Yang, Yongsheng Yu

**Affiliations:** 1 MIIT Key Laboratory of Critical Materials Technology for New Energy Conversion and Storage, School of Chemistry and Chemical Engineering, Harbin Institute of Technology, Harbin, 150001 Heilongjiang People’s Republic of China; 2 Space Environment Simulation Research Infrastructure, Harbin Institute of Technology, Harbin, 150001 People’s Republic of China

**Keywords:** Polymeric carbon nitride, Au nanoparticles, Pt single atoms, Photocatalytic H_2_ evolution, Broad-spectrum photocatalysts

## Abstract

Rationally designing broad-spectrum photocatalysts to harvest whole visible-light region photons and enhance solar energy conversion is a “holy grail” for researchers, but is still a challenging issue. Herein, based on the common polymeric carbon nitride (PCN), a hybrid co-catalysts system comprising plasmonic Au nanoparticles (NPs) and atomically dispersed Pt single atoms (PtSAs) with different functions was constructed to address this challenge. For the dual co-catalysts decorated PCN (PtSAs–Au_2.5_/PCN), the PCN is photoexcited to generate electrons under UV and short-wavelength visible light, and the synergetic Au NPs and PtSAs not only accelerate charge separation and transfer though Schottky junctions and metal-support bond but also act as the co-catalysts for H_2_ evolution. Furthermore, the Au NPs absorb long-wavelength visible light owing to its localized surface plasmon resonance, and the adjacent PtSAs trap the plasmonic hot-electrons for H_2_ evolution via direct electron transfer effect. Consequently, the PtSAs–Au_2.5_/PCN exhibits excellent broad-spectrum photocatalytic H_2_ evolution activity with the H_2_ evolution rate of 8.8 mmol g^−1^ h^−1^ at 420 nm and 264 μmol g^−1^ h^−1^ at 550 nm, much higher than that of Au_2.5_/PCN and PtSAs–PCN, respectively. This work provides a new strategy to design broad-spectrum photocatalysts for energy conversion reaction.

**Supplementary Information:**

The online version contains supplementary material available at 10.1007/s40820-023-01098-2.

## Highlights

- A hybrid co-catalysts system comprising Au nanoparticles (NPs) and PtSAs with different functions was constructed on the polymeric carbon nitride (PCN) surface by double-solvent method.
- For the dual co-catalysts, the Au NPs absorb relatively long-wavelength light to produce plasmonic hot-electrons, and the adjacent Pt single atoms (PtSAs) can trap the plasmonic hot-electrons effectively for H_2_ evolution.
- The PtSAs–Au_2.5_/PCN exhibits excellent broad-spectrum photocatalytic H_2_ evolution activity with the H_2_ evolution rate of 8.8 mmol g^−^^1^ h^−^^1^ at 420 nm and 264 μmol g^−^^1^ h^−^^1^ at 550 nm, respectively.

**Supplementary Information:**

The online version contains supplementary material available at 10.1007/s40820-023-01098-2.

## Graphical abstract

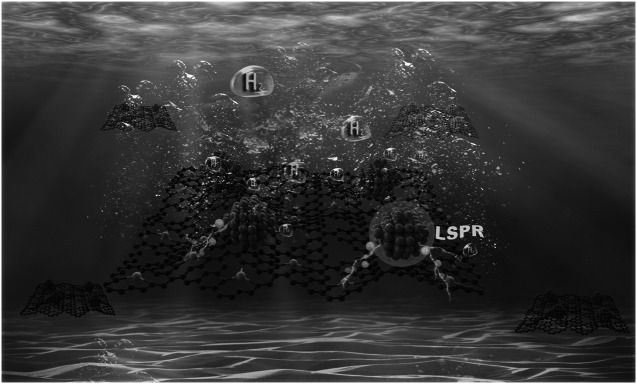

## Introduction

Photocatalytic water splitting to H_2_ has been regarded as a promising technology to convert renewable solar energy and address the global energy crisis [1–6]. Among various semiconductor photocatalysts, the polymeric carbon nitride (PCN) has attracted tremendous attention since its pioneering study in 2009 [7], owing to the nontoxicity, excellent chemical stability and facile preparation from abundant precursors [8–10]. However, the photocatalytic efficiency for pristine PCN is still too low because of the limited visible-light absorption and serious recombination of photogenerated electrons and holes [11]. To expand the visible-light harvest ability for full utilization of solar energy, a variety of strategies have been adopted to engineer the bandgap structure via elevating valance band (VB) and/or reducing the conductor band (CB) edge positions, including elemental doping [12–14], defect constructing [15–17] and crystallizing [18–20]. Unfortunately, the narrow-bandgap PCN with higher VB and/or lower CB edges generally shows lower redox abilities in compared with pristine PCN [21]. Hence, the irreconcilable relationship between extending intrinsic absorption edges and keeping redox ability of PCN is still a very hard nut to crack through simple bandgap engineering.

Recently, based on the localized surface plasmon resonance (LSPR) of metal nanoparticles (NPs, e.g., Au and Ag), integration of Au NPs into PCN has been widely explored to enable light absorption region up to the near infrared (NIR) without sacrificing its CB/VB redox ability [22–26]. As demonstrated by previous reports, the plasmonic Au NPs can be excited under specific light illumination to generate hot-electrons, which can jump over the Schottky barrier and flow into the CB of PCN when composing hybrid structures between Au NPs and PCN [22, 27]. Through tuning the shape, size and composition of Au NPs, the LSPR intensity and resonant photon wavelength of plasmonic metal can be engineered to harvest entire solar light [25]. For instance, the Au nanorods are assembled on PCN nanosheets to achieve near-infrared photocatalytic H_2_ evolution activity (63.1 μmol g^−1^ h^−1^) [28]. On the other hand, the Au NPs can also act as co-catalysts to trap photoelectrons from PCN via the Au/PCN Schottky interface [21, 29]. But it is a pity that the Au nanocrystals usually possess large size due to the nanostructure design and is inactive for H_2_ evolution reaction, resulting in poor photocatalytic efficiencies under the intrinsic absorption of PCN [28–30]. Furthermore, the photocatalytic activity of Au/PCN hybrid photocatalysts offered by the plasmonic effect is still mediocre due to the short diffusion length of hot-electrons and little active sites in PCN surface for H_2_ evolution reaction [27]. Hence, it is highly desirable as well as challenging to design plasmonic metal/PCN systems for achieving super photocatalytic activity under both short- and long-wavelength light.

In this work, we report the synergetic plasmonic Au NPs and atomically dispersed Pt single atoms (PtSAs) on PCN surface (PtSAs–Au_2.5_/PCN) to achieve broad-spectrum photocatalytic H_2_ evolution. In our design, well-dispersed Au NPs with a small size of about 4 nm were firstly anchored on PCN (Au_2.5_/PCN), in which tuning the particle size and metal–support interaction greatly improve the H_2_ evolution activity of Au NPs when served as the co-catalysts. The photocatalytic H_2_ evolution rate reaches 5.2 mmol g^−1^ h^−1^ at 420 nm for Au_2.5_/PCN. Besides, the Au NPs exhibit strong LSPR effect with broad absorption band from 500 to 800 nm. Subsequently, the PtSAs were incorporated into the Au_2.5_/PCN hybrid to further accelerate charge transfer and photoelectron capture, especially for plasmonic hot-electrons, which elevates the photocatalytic H_2_ evolution performance driven by LSPR effect over threefold. As a result, as-obtained PtSAs–Au_2.5_/PCN exhibits excellent photocatalytic H_2_ evolution activity under whole visible spectral region. The H_2_ evolution rate reach 8.8 mmol g^−1^ h^−1^ at 420 nm and 264 μmol g^−1^ h^−1^ at 550 nm, much higher than that of Au/PCN and PtSAs/PCN, respectively. This work provides a strategy to couple wide-bandgap semiconductor with plasmonic metal for broad-spectrum photocatalysis.

## Experimental Section

### Materials

Gold acid chloride trihydrate (HAuCl_4_∙3H_2_O, 99%), chloroplatinic acid (H_2_PtCl_6_∙6H_2_O, 99%), Potassium tetrachloroplatinate (K_2_PtCl_4_, 99%), n-hexane (AR) and triethanolamine (TEOA, AR) were all purchased from Aladdin and used without further purification. Urea was purchased from Tianli Chemical Reagent Co., Ltd. Deionized water was used for all the experiment.

### Synthesis of PCN, Au_x_/PCN and PtSAs–Au_2.5_/PCN

#### *Synthesis of PCN*

Briefly, the covered crucible with 15 g urea was placed into a muffle furnace and then heated at 550 °C for 4 h with a heating rate of 10 °C min^−1^ under a stable air atmosphere. After cooling down to room temperature, the resultant yellow products were milled into powder in a mortar, named as PCN.

#### ***Synthesis of Au***_***x***_***/PCN***

Au_x_/PCN was prepared by double-solvent method. Typically, 48.75 mg PCN was suspended in 15 mL n-hexane with vigorous stirring. 0.2 mL HAuCl_4_∙3H_2_O solution (12.5 mg mL^−1^) was added into the above solution with constant stirring. After consistently stirring at room temperature for 2 h, the resulting solution was then sonicated for another 1 h. Later, the resultant HAuCl_4_/PCN slurry sank down into the bottom of the beaker. Subsequently, the HAuCl_4_/PCN slurry was dried at 50 °C after decanting the supernatant and then milled into powder in a mortar. Finally, the powder was placed into a tube furnace and heated at 450 °C for 2 h under H_2_/Ar atmosphere to yield Au_x_/PCN. Au_*x*_/PCN hybrids with different loading of Au NPs were prepared by changing the amount of PCN and HAuCl_4_∙3H_2_O with controlled mass values (PCN/HAuCl_4_∙3H_2_O: 49.5/1, 48.75/2.5 and 47.5/5 mg), respectively. The products were therefore denoted as Au_x_/PCN (*x* = 1, 2.5 and 5, and corresponds to the wt% of Au NPs). For comparison, the Au_2.5_-PCN and Pt-PCN were prepared by traditional photo-deposition method. Typically, 48.75 mg PCN and 2.5 mg HAuCl_4_∙3H_2_O were dispersed in 15 mL H_2_O with ultrasonic blending for 30 min. Then, the suspension was evacuated to remove dissolved air completely and irradiated by a 300 W Xe lamp for 1 h to reduce the Au cations. After centrifugal separation, the obtained powder was named as Au_2.5_-PCN. The Pt-PCN was prepared by similar method with only changing the precursor.

#### ***Synthesis of PtSAs–Au***_***2.5***_***/PCN***

The PtSAs–Au_2.5_/PCN was prepared with similar to that Au_2.5_/PCN. Typically, 0.2 mL K_2_PtCl_4_ solution (5.3 mg mL^−1^) was added into the Au_2.5_/PCN suspension (49.5 mg Au_2.5_/PCN in 15 mL n-hexane) with vigorous stirring. After consistently stirring for 2 h, the resulting suspension was then sonicated for another 1 h. Subsequently, the K_2_PtCl_4_-Au_2.5_/PCN slurry was obtained by decanting the supernatant. After drying at 50 °C overnight, the K_2_PtCl_4_-Au_2.5_/PCN intermediate was milled into powder in a mortar. Finally, the powder was placed into a tube furnace and heated at 150 °C for 2 h under H_2_/Ar atmosphere to yield PtSAs–Au_2.5_/PCN. In addition, with changing the Au_2.5_/PCN as PCN, the PtSAs–PCN was successfully prepared.

### Photocatalytic H_2_ Evolution Tests

The photocatalytic H_2_ evolution experiments were performed using an on-line photocatalytic analysis system (Labsolar IIIAG, Beijing Perfectlight) with a quartz reactor. Ten milligram photocatalysts were dispersed in 100-mL TEOA (20%) aqueous solution. Then, the suspension was evacuated several times to completely remove dissolved air and then irradiated by a 300 W Xe lamp with various filters, such as cutoff filter (*λ* ≥ 420 nm) and band-pass filters (420, 450, 550, 600 and 650 nm). The amounts of H_2_ were collected and analyzed every hour by the gas chromatography equipped with a thermal conductive detector (TCD).

## Results and Discussion

### Synthesis and Characterizations of Aux/PCN

**Fig. 1:**
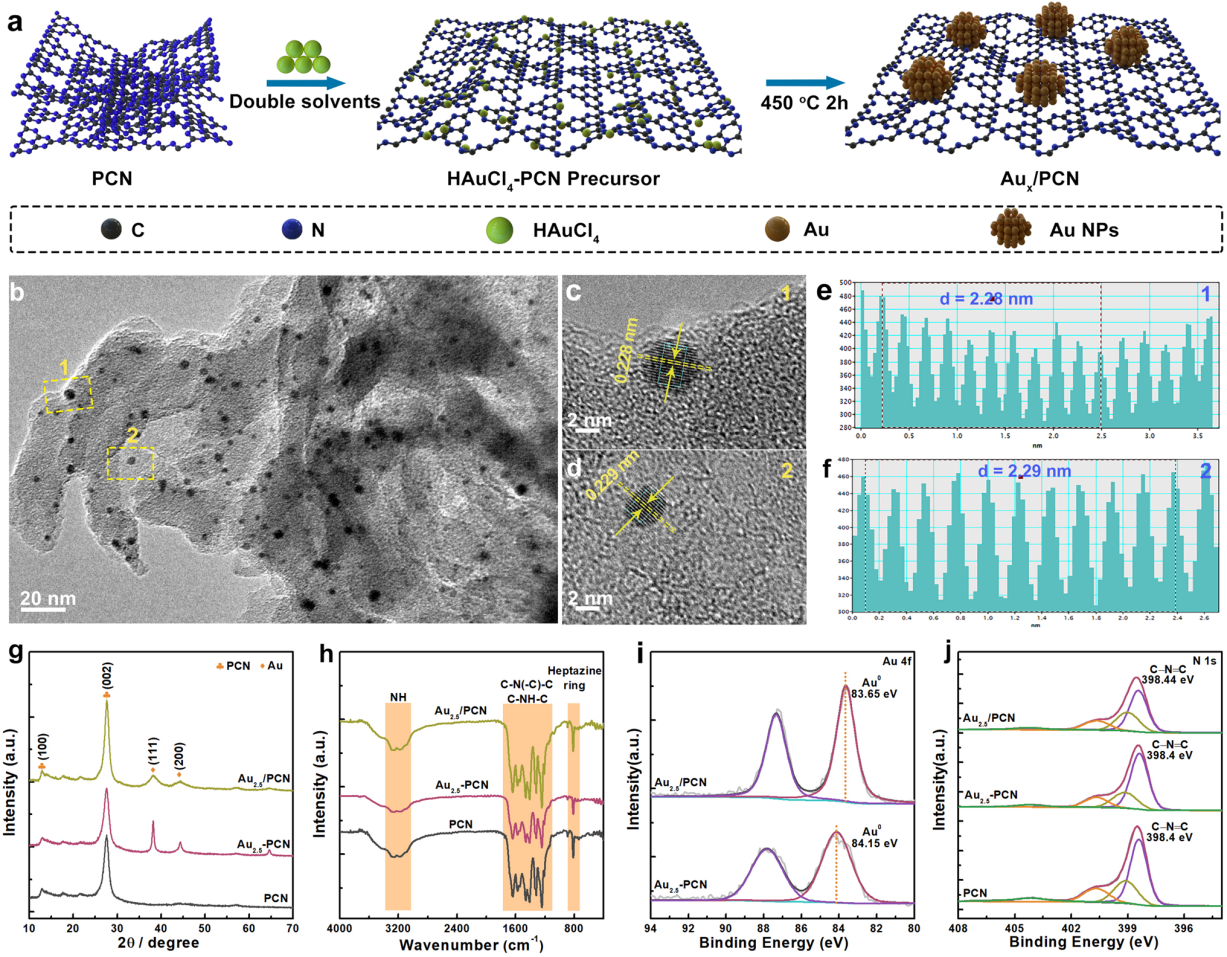
**a** Synthesis and structural characterizations of Au_*x*_/PCN photocatalysts. **a** Illustration of the preparation process. **b** TEM image of Au_2.5_/PCN. **c, d** High-resolution TEM images of Au_2.5_/PCN for the marked rectangular areas in **b** and **e and f** the corresponding Au intensity profile. **g** XRD patterns and **h** FTIR spectrums and high-resolution XPS spectra: **i** Au 4*f,* **j** N 1*s* for Au_2.5_/PCN and reference samples

The overall synthesis procedure of Au_*x*_/PCN with different Au loading amounts is illustrated in Fig. 1a. Typically, the HAuCl_4_/PCN intermediates were prepared by a double-solvent method with a subsequent heating treatment at 450 °C to obtain Au_*x*_/PCN (*x* = 1, 2.5 and 5, which represents the loading amount of Au NPs). As shown in Figs. 1b and S1, the transmission electron microscopy (TEM) images of Au_*x*_/PCN reveal that the Au NPs are distributed on PCN surface with the average size of about 4 nm (Fig. S2). Clear lattice fringes for select Au NPs in Au_2.5_/PCN sample (Fig. 1b) are observed in the high-resolution TEM images (Fig. 1c, d), and the lattice distance is calculated to be 0.228/0.229 nm (Fig. 1e, f), corresponding to the (111) planes of fcc structured Au. For comparison, 2.5 wt% of Au NPs were also loaded on PCN by universal photodeposition method (Fig. S3a, named as Au_2.5_-PCN), in which the average diameter of Au NPs is 13 nm (Fig. S3b), much larger than that of Au_2.5_/PCN. The size discrepancy of Au NPs in Au_2.5_/PCN and Au_2.5_-PCN is further demonstrated by the X-ray diffraction (XRD) patterns, in which significantly reduced full width at half maximum of (111) and (200) diffraction peaks can be found in Au_2.5_-PCN (Fig. 1g). Besides, the other diffraction peaks at 12.9° and 27.6° in theses samples can be assigned to (100) and (002) crystal planes of PCN, which corresponds to the repeating in-plane packing motif of heptazine units and interlayer stacking of aromatic segments, respectively [31]. This demonstrates that the loading of Au NPs does not destroy the crystalline structure of PCN. Moreover, in the Fourier transform infrared (FTIR) spectroscopy (Fig. 1h), similar characteristic vibrations at 810 cm^−1^, 1100 to 1700 cm^−1^ and 3100 to 3300 cm^−1^ exist in these samples, assigned to vibration of tri-s-triazine units, stretching vibration modes of C-N heterocycles as well as O–H and N–H stretching vibration [32], also suggesting that there is no variation of tri-s-triazine molecular skeleton for PCN.

The X-ray photoelectron spectroscopy (XPS) was performed to investigate the surface compositions and chemical states of PCN and Au NPs-modified PCN samples. As displayed in Fig. S4a, the survey spectra of Au_2.5_/PCN and Au_2.5_-PCN show that a new peak appears at around 84 eV correspond to Au 4*f* signals, which indicates the successful deposition of Au NPs on the PCN surface. For the Au element, compared to those of Au_2.5_-PCN, the Au 4*f* peaks of Au_2.5_/PCN show apparent shift toward lower energy positions by ca. 0.5 eV (Fig. 1i), indicating the increased electron density of Au NPs in Au_2.5_/PCN sample [33]. The enhanced electron transfer from PCN to Au NPs in Au_2.5_/PCN confirms a strong electronic metal–support interaction (EMSI) between PCN and Au NPs [34], which is benefit to photogenerated electron transfer at Au-PCN Schottky interface. While for C and N signals, the alteration of peak position is negligible between Au_2.5_/PCN and reference samples, probably due to the much larger mass content of PCN than that of Au NPs. Besides, the characteristic peaks for triazine units are still overwhelming majority after the loading of Au NPs (Figs. S4b and 1j), including N–C = N *sp*^2^ carbon (ca. 288 eV), C–N = C *sp*^2^ nitrogen (ca. 398.4 eV) and N-(C)_3_ *sp*^3^ nitrogen (ca. 399 eV) [35], which is in consistent with the above XRD and FTIR results.

### Photocatalytic Properties of Au_x_/PCN

**Fig. 2:**
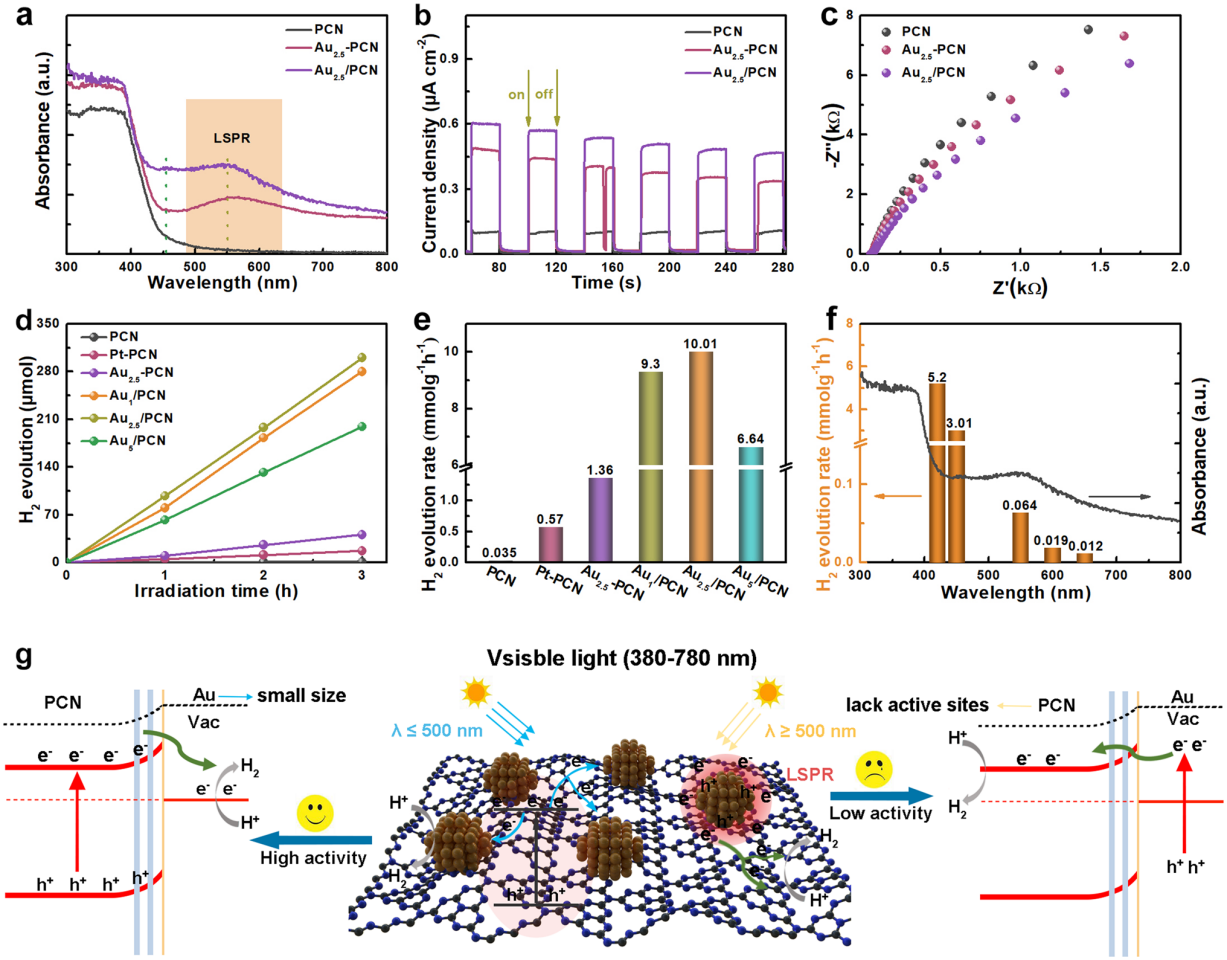
Optics and photocatalytic properties of Au_*x*_/PCN photocatalysts. **a** UV–vis DR spectra of PCN, Au_2.5_-PCN and Au_2.5_/PCN. **b** TPR density and **c** EIS Nyquist plots of PCN and Au_2.5_/PCN. **d** Time-dependent course of H_2_ evolution and **e** comparison of the photocatalytic H_2_ evolution rate for Au_2.5_/PCN and reference samples. **f** Photocatalytic H_2_ evolution under various wavelength for Au_2.5_/PCN. **g** The photocatalytic mechanism Au_2.5_/PCN under different visible-light regions

The ultraviolet–visible diffuse reflectance (UV–vis DR) spectra of these as-prepared samples were acquired to characterize its optical properties (Fig. 2a). As expected, the Au-containing samples show similar absorption feature with pure PCN under UV and short-wavelength visible light, and the absorption edges are around 460 nm without obvious shift, corresponding to the unaltered bandgap energy for PCN (about 2.69 eV) [30]. Compared with pure PCN, an extra broad absorption band appears in the enlarged absorption spectrum of Au-containing samples with a range of 500–800 nm, which originates from the LSPR effect of Au NPs [36–38]. Hence, the incorporation of Au NPs could improve the overall light absorption of Au_2.5_/PCN, especially in the long-wavelength visible light. In addition, the LSPR absorption of Au_2.5_/PCN with slightly blue-shift is much stronger than that of Au_2.5_-PCN, which is mainly attributed to the different size and surrounding electric environment of Au NPs [21]. Photochemical tests were carried out to investigate the charge transfer behaviors in Au_2.5_/PCN, including transient photocurrent response (TPR) and electrochemical impedance (EIS) spectra (Fig. 2b, c). Compared to PCN and Au_2.5_-PCN, the Au_2.5_/PCN exhibits highest photocurrent density and smallest impedance arc radius, meaning significantly enhanced electron transfer kinetics in Au_2.5_/PCN. This highlights the superiority of strong EMSI at Au-PCN interface for charge mobility.

The photocatalytic water splitting to H_2_ over Au_2.5_/PCN and reference samples was evaluated under visible-light irradiation with TEOA as sacrificial reagent via an on-line photocatalytic analysis system (Labsolar IIIAG, Beijing Perfectlight). As shown in Fig. 2d, the pure PCN shows mediocre photocatalytic H_2_ evolution activity with the rate of only 35 μmol g^−1^ h^−1^ (Fig. 2e) under visible light, and its H_2_ evolution rate increases to 1.36 mmol g^−1^ h^−1^ with the loading of Au NPs on PCN surface as co-catalysts via in situ photodeposition way, probably due to the increase of activity sites for H_2_ evolution as well as enhanced photogenerated carrier separation. Moreover, the photocatalytic H_2_ evolution activity is further elevated for Au_x_/PCN with the regulation of Au NPs size and Au-PCN interface, and the highest H_2_ evolution rate reaches up to 10.01 mmol g^−1^ h^−1^ after optimizing the Au loading amount, approximately 7.4 and 17.5 times higher than that of Au_2.5_-PCN and Pt-PCN.

To further investigate the fundamental role of Au NP in boosting photocatalytic activity of PCN, H_2_ evolution measurements were conducted under irradiation of different individual wavelength. As shown in Fig. 2f, the Au_2.5_/PCN displays excellent photocatalytic H_2_ evolution activity at the intrinsic absorption of PCN (about 400–450 nm). The H_2_ evolution rate achieves 5.2 mmol g^−1^ h^−1^ at 420 nm, suggesting that Au NPs indeed acts as the co-catalysts to trap photogenerated electrons for H_2_ evolution reaction (Fig. 2g) [29]. In the photocatalytic process, the strong EMSI can accelerate the electron transfer through Au-PCN Schottky interface and the Au NPs with small size can provide abundant active sites for H_2_ evolution. On the other hand, under the long-wavelength light irradiation (about 500–780 nm), the Au_2.5_/PCN also exhibits photocatalytic H_2_ evolution activity, which originates from the LSPR effect of Au NPs, that is, the plasmonic hot-electrons jump over the Schottky barrier and inject into the CB of PCN for H_2_ evolution (Fig. 2g) [22, 29]. The reproducible photocurrent at 550 nm light irradiation (Fig. S5) further confirms the transfer process of hot-electrons. However, the photocatalytic efficiency purely offered by plasmonic hot electrons is quite poor with the H_2_ evolution rate of only 64 μmol g^−1^ h^−1^ at 550 nm. The major reason for this situation is the short diffusion length of hot-electrons and the lack of active sites near the Au NPs to trap these electrons for H_2_ evolution.

### Synthesis and Characterizations of PtSAs–Au_2.5_/PCN

**Fig. 3:**
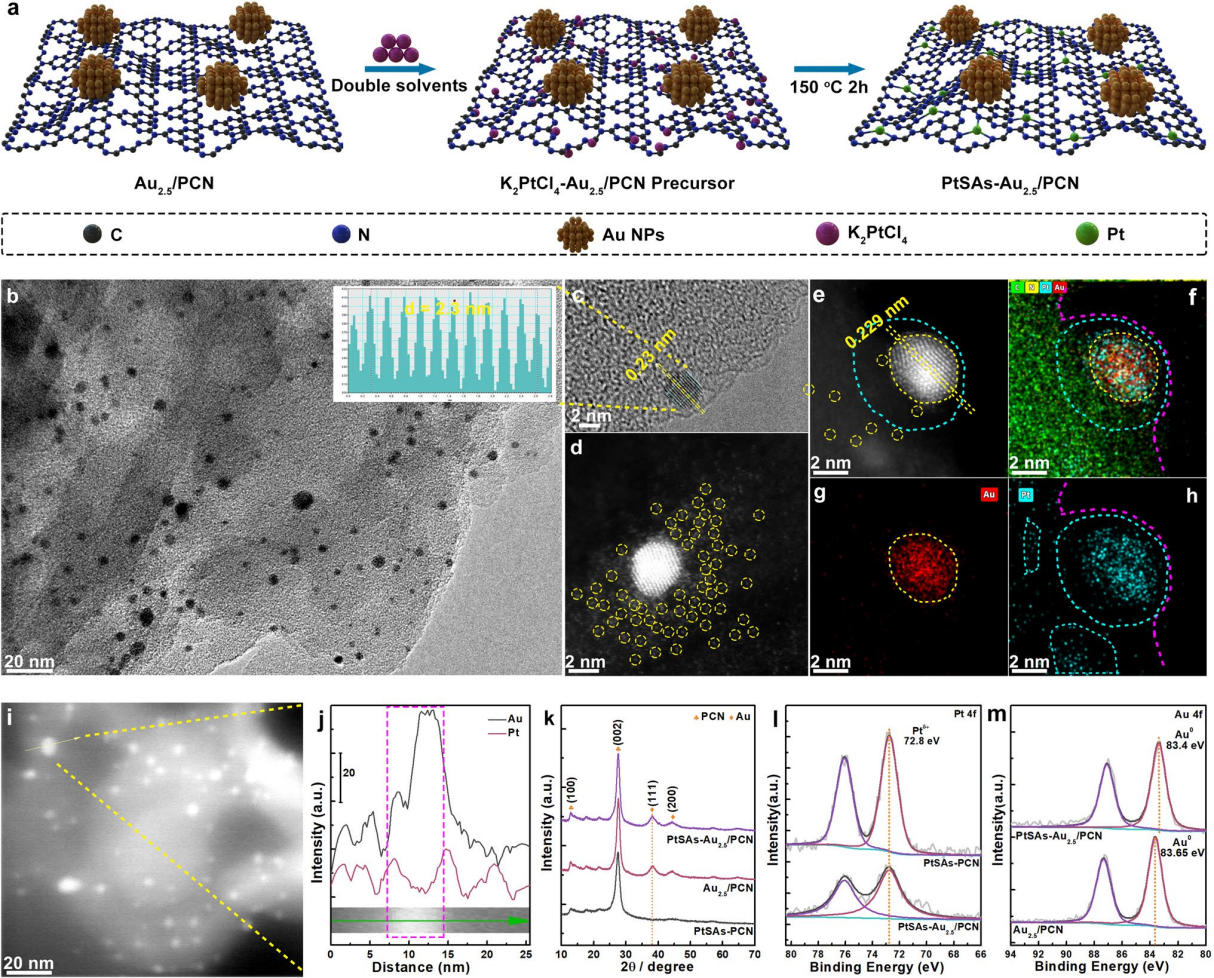
Synthesis and structural characterizations of PtSAs–Au_2.5_/PCN photocatalysts. **a** Illustration of the preparation process. **b** TEM and high-resolution TEM (insertion) images of PtSAs-Au_2.5_/PCN. **c** HAADF-STEM, **d** enlarged HAADF-STEM images, **e–g** EDS elemental mappings and **h, i** line-scan EDS analysis across a single Au NPs for PtSAs–Au_2.5_/PCN. **j** XRD patterns and high-resolution XPS spectra of **k** Au 4*f*, **i** Pt 4*f* for PtSAs-Au_2.5_/PCN and reference samples

Based on the above analysis, to further improve the photocatalytic activity, the core concept is to incorporate new electron acceptors into PCN plane near the Au NPs for efficient plasmonic hot-electron transfer and capture [39]. Metal SAs with sub-nanometer size have been widely designed as the photocatalytic H_2_ evolution sites to trap photoexcited electrons from PCN [34, 40–42]. Based on its unique size effect, engineering the metal SAs adjacent to Au NPs with several nanometer distance may be an effective way for plasmonic hot-electron capture. As a proof-of-concept demonstration, starting from the Au_2.5_/PCN sample, the PtSAs are further deposited on PCN surface with similar synthetic process to that of Au_2.5_/PCN (Fig. 3a, denoted as PtSAs–Au_2.5_/PCN). As presented in Fig. 3b, the Au NPs are still well dispersed on the surface of PCN with the average size of 4.1 nm (Fig. S6), slightly larger than that of Au_2.5_/PCN. The high-resolution TEM and HAADF-TEM images show clear lattice fringes with a spacing of 0.23/0.229 nm, which could be attributed to the *fcc* Au (111) plane (Fig. 3c, e). Moreover, there are numerous bright dots around Au NPs with several nanometer distances (Fig. 3d and Fig. S7), corresponding to the atomically dispersed PtSAs. The elemental distribution is investigated by the energy-dispersive spectroscopy (EDS) elemental mappings and line-scan EDS analysis (Fig. 3e-j). The Au element is contained within the nanocrystalline, while the Pt element distributes across the whole PCN region, especially in the surrounding of Au NPs. The results demonstrate that partial PtSAs are indeed formed near the Au NPs in PtSAs–Au_2.5_/PCN. For comparison, the PtSAs were also anchored on the surface of PCN (named as PtSAs–PCN, Fig. S8).

The crystal structures of as-prepared samples were investigated by powder XRD. As presented in Fig. 3k, the characteristic peaks of PCN at 12.9° and 27.6° exist in these samples without obvious variation, meaning no destruction of crystalline structure for PCN [43, 44], which is in consistent the FTIR results (Fig. S9). Besides, there are other two distinct diffraction peaks at about 38.2° and 44.4° in the XRD patterns of Au_2.5_/PCN and PtSAs–Au_2.5_/PCN, indexed to the typical (111) and (200) planes of *fcc* structured Au. And no peak shift can be found between Au_2.5_/PCN and PtSAs–Au_2.5_/PCN, excluding the existence of AuPt alloying during the deposition of PtSAs. The chemical state of surface atoms was analyzed by the XPS (Figs. 3l, m and S10, S11). Obviously, only Pt^δ+^ peaks (72.8 eV, Pt 4*f*) are observed for PtSAs–Au_2.5_/PCN and PtSAs-PCN (Fig. 3k), again excluding the formation of Pt or AuPt NPs in PtSAs–Au_2.5_/PCN sample. As for the Au 4*f* signals in Fig. 3l, the Au 4*f*_7/2_ peaks at about 83.5 eV for Au_2.5_/PCN and PtSAs-Au_2.5_/PCN can be assigned to metallic Au^0^ species. Compared to that of Au_2.5_/PCN, Au 4*f*_7/2_ peak of PtSAs–Au_2.5_/PCN shows a negative shift by ca. 0.25 eV, confirming more electron transfer from PCN to Au NPs after the deposition of PtSAs [32]. This strongly indicate an enhanced EMSI between PCN and Au NPs. While for the C 1*s* and N 1*s* signals, the peak shift is virtually invisible between Au_2.5_/PCN and PtSAs–Au_2.5_/PCN, probably originating from a fairly low mass content of Au NPs in PtSAs–Au_2.5_/PCN.

**Fig. 4:**
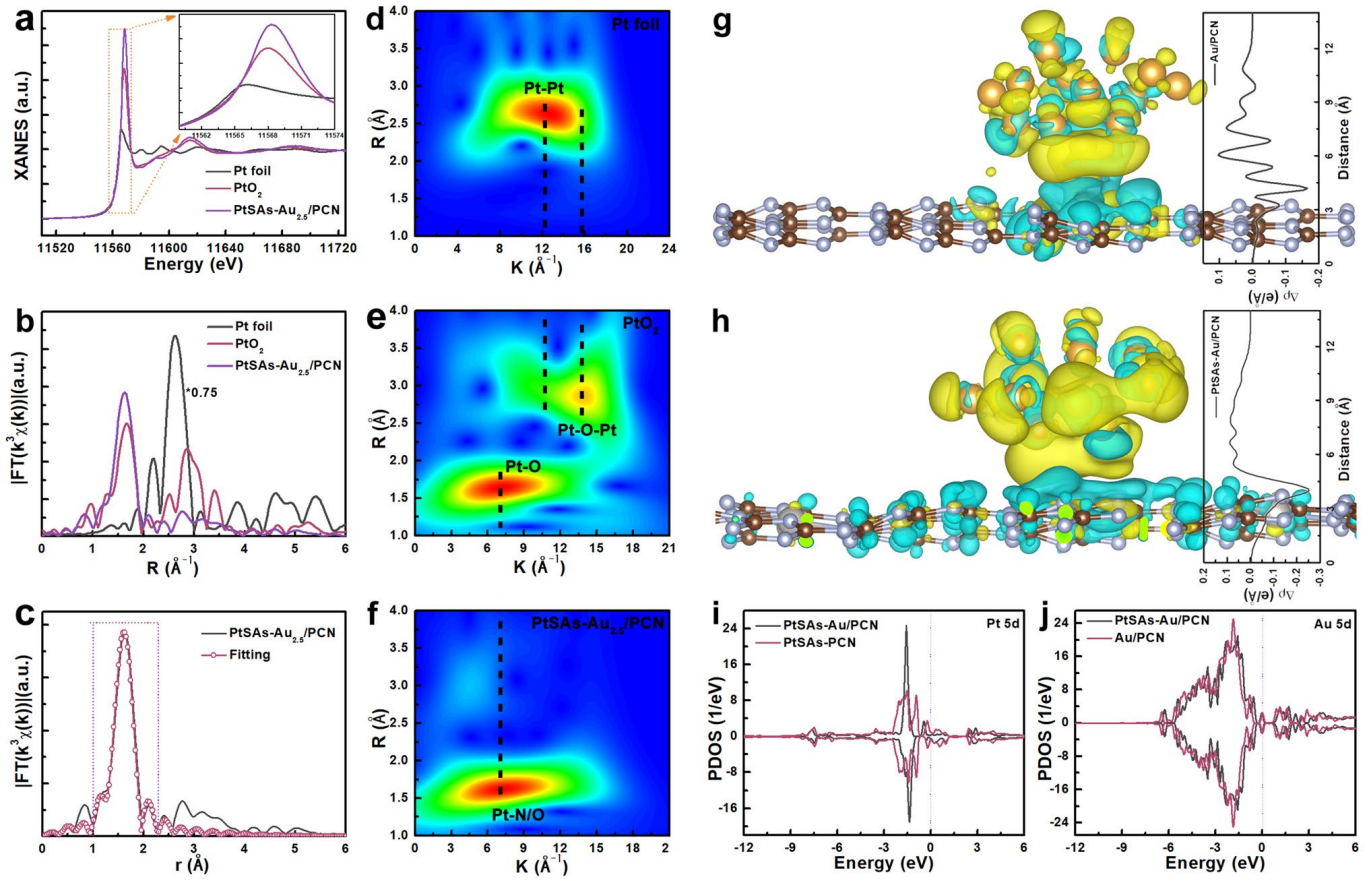
Surface properties of PtSAs-Au_2.5_/PCN photocatalysts. **a** Pt L_3_-edge XANES spectra and the corresponding FT k^3^-weighted spectra at **b** R space. **c** Pt EXAFS fitting curves of PtSAs–Au_2.5_/PCN at R space. WT of **d** Pt foil, **e** PtO_2_ and **f** PtSAs–Au_2.5_/PCN. Difference charge density analysis and two-dimensional average difference charge density diagrams (insertion) for **g** Au/PCN and **f** PtSAs-Au/PCN model, yellow and cyan represent electron accumulation and depletion, respectively. **i, j** The calculated projected density of states (PDOS) for PtSAs-PCN, Au/PCN and PtSAs-Au/PCN model

To further investigate the valence state and local atomic structure of Pt sites in PtSAs–Au_2.5_/PCN, the X-ray absorption spectroscopy (XAS) measurements were taken to acquire the X-ray absorption near-edge structure (XANES) and extended X-ray fine structure (EXAFS) spectroscopy. XANES results in Fig. 4a show that the Pt L_3_-edge white line intensity of PtSAs–Au_2.5_/PCN is higher than that of PtO_2_, meaning high oxidation state of Pt sites, which is consistent with the high-resolution XPS spectra Pt 4*f*. The Fourier transformed (FT) k_3_-weighted EXAFS spectra of PtSAs–Au_2.5_/PCN at R space (Fig. 4b) reveals a sharp peak at near 1.6 Å, which can be attributed to the Pt–N/O scattering, testifying the atomically dispersed PtSAs on PCNS surface. Moreover, the Morlet wavelet transforms (WT) of Pt L_3_-edge EXAFS spectra further confirm the existence of Pt sites in PtSAs–Au_2.5_/PCN (Fig. 4d-f), which has the maximum WT intensity at about *R* = 1.6 Å, *k* = 7.1 Å^−1^ with compared to the Pt–Pt bond in Pt foil (*R* = 2.6 Å, *k* = 13.1 Å^−1^) [32, 34]. The EXAFS fitting spectrum of Pt foil and PtSAs–Au_2.5_/PCN was performed to probe quantitative atomic structure of Pt sites as shown in Figs. 4c and S12, S13. The obtained R-space fitting curves of PtSAs–Au_2.5_/PCN matches well with the experimental data at the range from 1.0 to 2.4 Å, demonstrating the formation of Pt-N_6_ bonds (Table S1). Hence, the above results provide a quantitative illustration for the existence of PtSAs near Au NPs.

To identify the influence of PtSAs on the electronic structure of PtSAs–Au_2.5_/PCN, density functional theory (DFT) calculations were performed on the basis of the above experimental results. The differential charge density diagrams reveal that the electron migration occurs from PCN to Au NPs through the intimate interface for Au/PCN (Figs. 4g and S14a), and the obviously enhanced electron transfer can be observed at the Au-PCN interface with the introduction of PtSAs (Figs. 4h andS14b) [45]. Moreover, the two-dimensional average difference charge density diagrams (insertion of Fig. 4g, h) clearly show that the maximum value of ∆ρ increases from − 0.17 e Å^−1^ for Au/PCN to − 0.3 e Å^−1^ for PtSAs–Au_2.5_/PCN, meaning that the Au NPs acquire more electrons from PCN in PtSAs–Au_2.5_/PCN. This further confirms the strong electronic metal–support interaction between Au NPs and PCN. On the other hand, in the calculated PDOS profiles (Fig. 4i, j), the decoration of PtSAs into the triangular sub-nanometer cavity near Au NPs greatly varies the energy levels and spatial distributions of Pt 5*d* and Au 5*d* orbitals as compared with that of isolated PtSAs or Au NPs, making the Au 5*d* DOS peaks slightly shift toward the Fermi level, which probably originates from the electron movement between PtSAs/Au NPs and PCN substrate. This not only accelerates the photogenerated charge transfer but also activate the Au NPs for H_2_ evolution reaction.

### Photocatalytic Properties of PtSAs–Au_2.5_/PCN

**Fig. 5:**
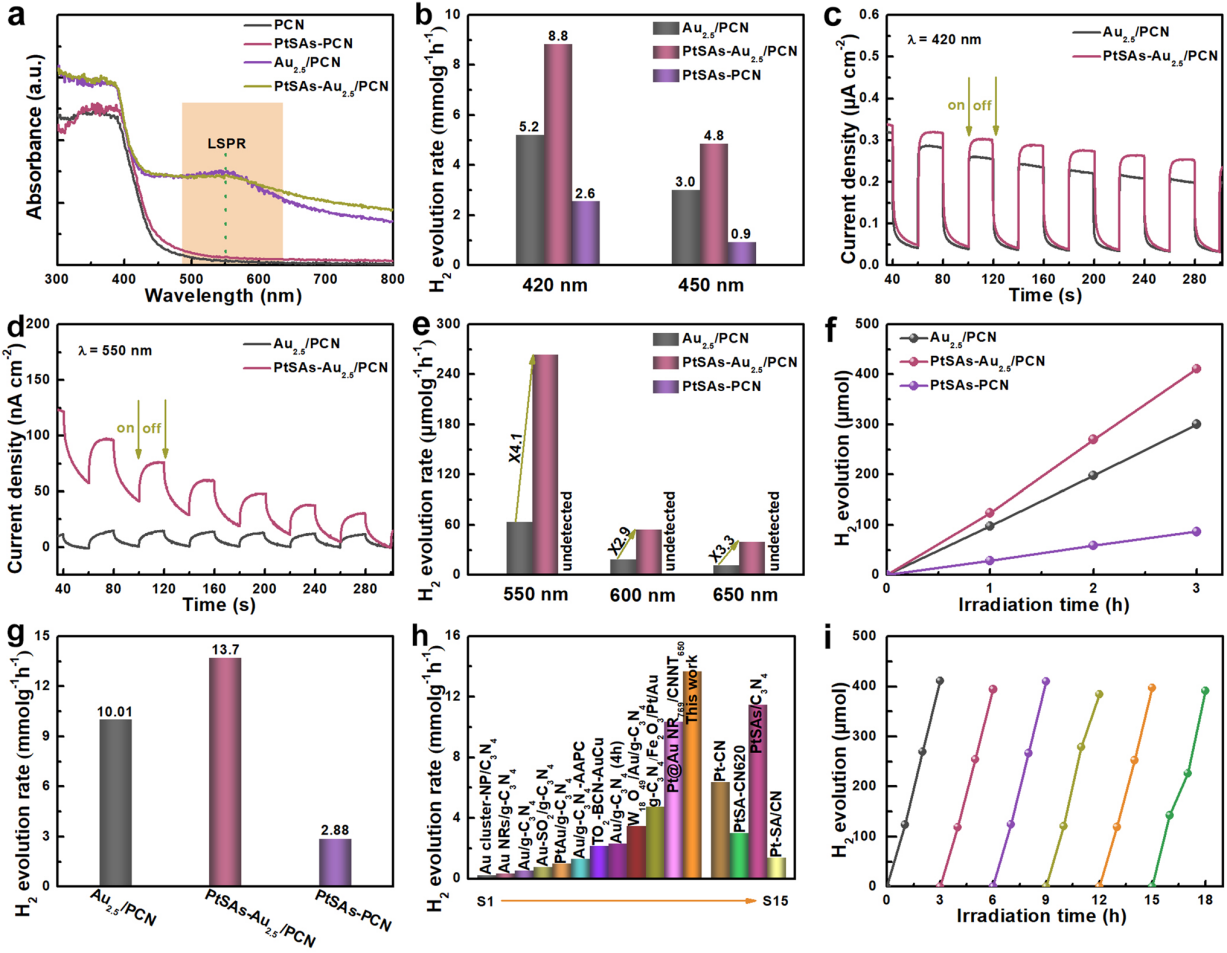
Optics and photocatalytic properties of plasmonic PtSAs–Au_2.5_/PCN. **a** UV–vis DR spectra of PtSAs–PCN, Au_2.5_/PCN and PtSAs–Au_2.5_/PCN. Comparison of the photocatalytic H_2_ evolution rate between PtSAs-Au_2.5_/PCN and reference samples under different wavelength light irradiation: **b** short-wavelength visible light and **e** long-wavelength visible light. TPR density of PtSAs–Au_2.5_/PCN and reference samples under different wavelength light irradiation: **c** *λ* = 420 nm, **d** *λ* = 550 nm and. **f** Time-dependent course of H_2_ evolution under visible light (*λ* ≥ 420 nm). **g** Comparison of the photocatalytic H_2_ evolution rate between PtSAs–Au_2.5_/PCN and reference samples as well as **h** previous Au-based PCN plasmonic photocatalysts in table S1. **i** Cycling photocatalytic test for PtSAs-Au_2.5_/PCN

Upon the successful deposition of PtSAs, we set out to explore that whether the PtSAs can facilitate plasmonic hot-electron transfer and improve photocatalytic H_2_ evolution. Firstly, the optical response properties of samples were characterized by the UV–vis DR spectra (Fig. 5a), the PtSAs–Au_2.5_/PCN shows similar light absorption to that of Au_2.5_/PCN with clear plasmonic peak at around 550 nm, which indicates no obvious variation of the LSPR effect of Au NPs after the introduction of PtSAs. Subsequently, to look into the role of PtSAs, the H_2_ evolution and TPR measurements of PtSAs–Au_2.5_/PCN and reference samples were conducted under different wavelength light irradiation as shown in Fig. 5b-e. Under the intrinsic absorption of PCN, the PtSAs-PCN shows moderate photocatalytic H_2_ evolution activity with the rate of 2.6/0.9 mmol g^−1^ h^−1^ at 420/450 nm (Fig. 5b), suggesting that the PtSAs can act as co-catalysts to trap the photoelectrons from PCN. Strikingly, the photocatalytic H_2_ evolution rate of PtSAs-Au_2.5_/PCN increases to 8.8/4.8 mmol g^−1^ h^−1^ under illumination of 420/450 nm light, much higher than that of Au_2.5_/PCN or PtSAs–PCN and even larger than the sum of H_2_ production rates of the two samples, which indicates the synergistic effect between Au NPs and PtSAs. Moreover, the enhanced photocurrent intensity further highlights the role of PtSAs on accelerating carrier separation in PtSAs–Au_2.5_/PCN (Fig. 5c). On the other hand, the photocurrent of PtSAs–Au_2.5_/PCN is enhanced by about 2.5 times relative to Au_2.5_/PCN under 550 nm light irradiation (Fig. 5d). This discrepancy is much larger than that of same photocatalysts at 420 nm (about 1.2). It suggests that the PtSAs near the Au NPs can effectively trap the injected plasmonic hot-electrons for H_2_ evolution, which may be responsible for the significantly enhanced photocatalytic performance of PtSAs–Au_2.5_/PCN under LSPR region. As displayed in Fig. 5e, the photocatalytic H_2_ evolution rate of PtSAs–Au_2.5_/PCN reaches up to 264 μmol g^−1^ h^−1^ at the strongest plasmonic-coupling wavelength (550 nm) and remains 55 μmol g^−1^ h^−1^ at 650 nm, much higher than that of Au_2.5_/PCN (about 4.1 times at 550 nm). In the meantime, the amount of photocatalytic H_2_ evolution is undetected for PtSAs–PCN at 550/600/650 nm, excluding the possibility of PCN as the source of photogenerated electrons. These results evidently demonstrate that the enhanced photocatalytic H_2_ evolution activity of PtSAs–Au_2.5_/PCN under LSPR region is attributed to more effective utilization of plasmonic hot-electrons.

Hence, benefiting from the photoelectron trapping effect of PtSAs, the PtSAs–Au_2.5_/PCN exhibits dramatically enhanced photocatalytic H_2_ evolution performance at both the intrinsic absorption of PCN and the LSPR region. As a result, the photocatalytic H_2_ evolution rate of PtSAs–Au_2.5_/PCN is significantly enhanced with compared to Au_2.5_/PCN (Fig. 5f), achieving 13.7 mmol g^−1^ h^−1^ under visible-light region (Fig. 5g), which outperforms a broad range of Au-based PCN plasmonic photocatalysts (Fig. 5h and Table S2). The highest photocurrent response and smallest impedance arc radius further reveal the improved electron transport for H_2_ evolution in PtSAs–Au_2.5_/PCN (Fig. S15). In addition, the PtSAs–Au_2.5_/PCN presents high catalytic stability without obvious decay after 18 h catalytic experiments (Figs. 5i and S16).

**Fig. 6:**
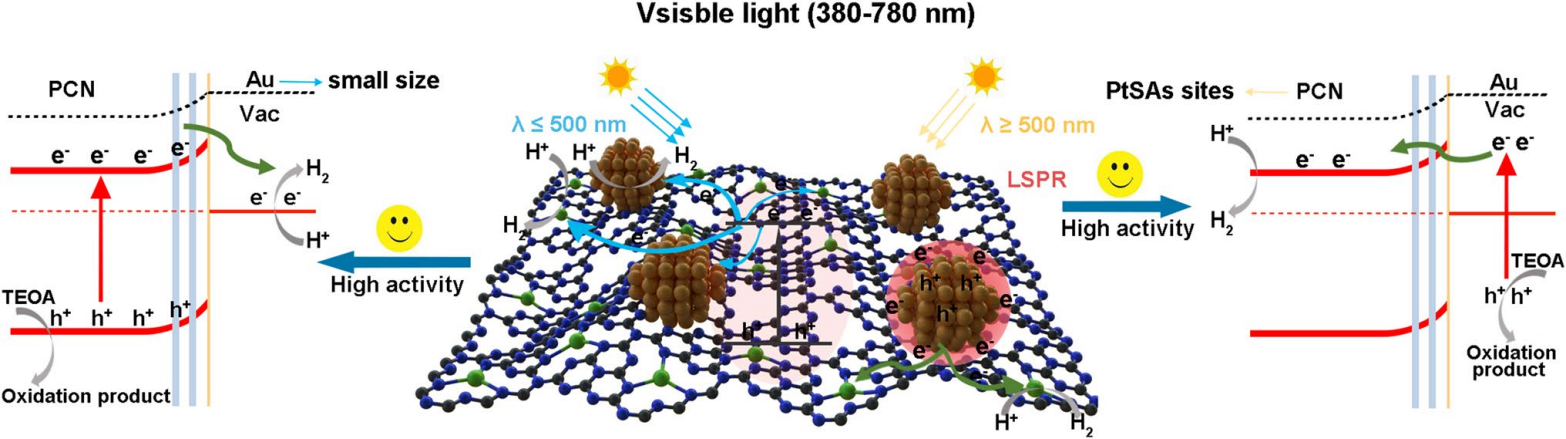
Photocatalytic mechanism of PtSAs–Au_2.5_/PCN under visible light

Based on the above discussions, the enhanced photocatalytic H_2_ evolution activity of PtSAs–Au_2.5_/PCN system under whole visible-light irradiation should be ascribed to two mechanisms as illustrated in Fig. 6. Exposure to short-wavelength light, the PCN is photoexcited to generate electrons on the conduction band due to its intrinsic absorption, and the synergistic Au NPs and PtSAs trap these photoelectrons for H_2_ evolution. On the other hand, the Au NPs absorb relatively long-wavelength light to produce plasmonic hot-electrons, which can jump over the Schottky barrier and rapidly transfer to the PtSAs for H_2_ evolution. Therefore, the introduction of PtSAs greatly improve the broad-spectrum photocatalytic H_2_ evolution activity, especially in the plasma response range of Au NPs.

## Conclusions

In summary, we have successfully designed and fabricated Au NPs and PtSAs on PCN surface to extend the visible-light absorption range as well as expedite plasmonic hot-electron injection and transfer for broad-spectrum photocatalytic H_2_ evolution. Thanks to the synergetic Au NPs and PtSAs, the as-prepared PtSAs-Au_2.5_/PCN exhibits excellent photocatalytic performance under whole visible-light irradiation. The H_2_ evolution rate reaches 13.7 mmol g^−1^ h^−1^ under the visible light and 264 μmol g^−1^ h^−1^ at 550 nm, much higher than that of Au/PCN and PtSAs/PCN, respectively. Detailed investigation has been carried out to reveal the enhancement photocatalytic mechanism. Briefly, the PCN is photoexcited under short-wavelength light to generate electrons on the conduction band, and the synergistic Au NPs and PtSAs trap these photoelectrons for H_2_ evolution. While the Au NPs absorb relatively long-wavelength light to produce plasmonic hot-electrons, which can jump over the Schottky barrier and rapidly transfer to the PtSAs for H_2_ evolution. This work opens up a new avenue to couple wide-bandgap semiconductor with plasmonic metal for broad-spectrum photocatalytic energy conversion applications.

### Supplementary Information

Below is the link to the electronic supplementary material.

Supplementary file1 (PDF 1871 KB)
