# Supplementary material for: Improved Plasmonic Hot-Electron Capture in Au Nanoparticle/Polymeric Carbon Nitride by Pt Single Atoms for Broad-Spectrum Photocatalytic H2 Evolution
Source: Nanomicro Lett. 2023 May 20;15:129. doi: 10.1007/s40820-023-01098-2 (PMC10199823; doi:10.1007/s40820-023-01098-2)
Supplement: Supplementary file 1 — Supplementary file1 (PDF 1871 KB) [file 40820_2023_1098_MOESM1_ESM.pdf]

Supporting information for

Improved Plasmonic Hot-Electron Capture in Au Nanoparticle/Polymeric Carbon Nitride by Pt Single Atoms for Broad-Spectrum Photocatalytic H_2 Evolution

Manyi Gao[1], Fenyang Tia[1], Xin Zhang[1], Zhaoyu Chen[2], Weiwei Yang[1,*] and Yongsheng Yu[1,*]

[1]MIIT Key Laboratory of Critical Materials Technology for New Energy Conversion and Storage, School of Chemistry and Chemical Engineering, Harbin Institute of Technology, Harbin, Heilongjiang 150001, P. R. China

[2]Space Environment Simulation Research Infrastructure, Harbin Institute of Technology, Harbin 150001, P. R. China

*Corresponding authors. E-mail: ysyu@hit.edu.cn (Yongsheng Yu), yangww@hit.edu.cn (Weiwei Yang)

S1 Characterization

X-ray diffraction (XRD) patterns were collected on a PAN analytical X'Pert Powder diffractometer using Cu Kα radiation (λ = 1.5418 Å) with a scan rate of 5 °min-1. Transmission electron microscopy (TEM) images were acquired on a JEM-1400 microscope operating at 100 kV (JEOL Ltd). High resolution TEM (HRTEM) images were obtained on a JEM-2100 with an accelerating voltage of 200 kV. The Fourier transform infrared (FT-IR) spectra were acquired on a Nicolet iS5 FT-IR spectrometer. X-ray photoelectron spectroscopy (XPS) measurements were performed on a Thermo Scientific ESCA Lab 250 Xi with monochromic Al Kα radiation (VG, USA). UV-visible absorption spectra were recorded on a Perkin Elmer Lambda 35 spectrometer.

S2 XAFS Measurements and Data Analysis

The Pt L_3-edge XAS data were collected the beamline BL10 in Spring-8. The typical energy of the storage ring was 2.5 GeV. The hard X-ray was monochromatized with Si (111) double-crystal monochromator. The as-prepared samples were measured by pressing them into a 14 mm diameter disc. The acquired EXAFS data were processed according to the standard procedures using the ATHENA module implemented in the IFEFFIT software packages. The pristine data were pre-treated by subtracting the post-edge background and normalizing to obtain the k^3-weighted EXAFS spectra. Subsequently, k^3-weighted $x(k)$ data of Pt L_3-edge were Fourier transformed to R space using a hanning windows (dk = 1.0 $Å^{-1}$) to separate the EXAFS contributions from different coordination shells. To obtain the quantitative structural parameters around Pt atoms, quantitative curve parameter fitting was performed at R space using the ARTEMIS module of IFEFFIT software packages. The K ranges for PtSAs-$Au_{2.5}$/PCN and Pt foil are 2.5-12.4 and 3-16.2 Å, respectively, while the R ranges are 1-2.4 Å for PtSAs-$Au_{2.5}$/PCN and 1-3 Å for Pt foil.

S3 DFT Calculations

Density Functional Theory (DFT) calculations were carried out to simulate the geometry structures and electronic properties of the sample based on the Vienna Ab initio Simulation Package using the PBE exchange-correlation function. The interaction between valence electrons and the ionic core was described by the PAW pseudo-potential. Three models were built to simulate the PtSAs-PCN, $Au_{2.5}$/PCN and PtSAs-$Au_{2.5}$/PCN, named as PtSAs-PCN, Au/PCN and PtSAs-Au/PCN, respectively. The geometry structures were optimized with the cut off of 400 eV. All the atoms in the model were allowed to adjust until the magnitude of all residual forces was less than 0.02 eV $Å^{-1}$. The geometry optimization and the PDOS was were calculated by the cutoff energy of 400 eV and the Monkhorst-Pack k-point mesh of 3×3×1.

S4 Photoelectrochemical Measurements

Photochemical tests were performed on a CHI660E electrochemical workstation in 0.2 M Na_2SO_4 solution. In the standard three-electrode system, an ITO glass covered with the as-prepared samples served as the working electrodes, a Pt wires and Ag/AgCl (saturated KCl) was used as counter electrode and reference electrode, respectively. For the working electrodes, 2 mg of the as-prepared samples was mixed with 500 μL ethanol/Nafion solution (9:1), followed by sonicating for 30 min to obtain a slurry. Then 20 μL of the homogeneous ink was dropped onto ITO glass with an area of about 1 cm^2 and dried in air. Subsequently, the working electrodes were heated at 80 °C for 2 h to eliminate ethanol. Electrochemical impedance spectroscopy (EIS) spectra were recorded under an AC perturbation signal of 50 mV over the frequency range from 100 KHz to 1 Hz.

S5 Supplementary Figures and Tables

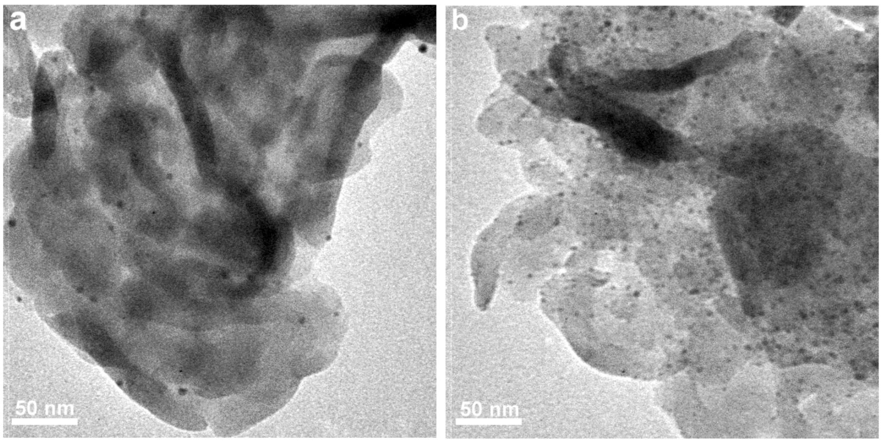

Fig. S1 TEM images of Au_1/PCN (**a**) and Au_5/PCN (**b**)

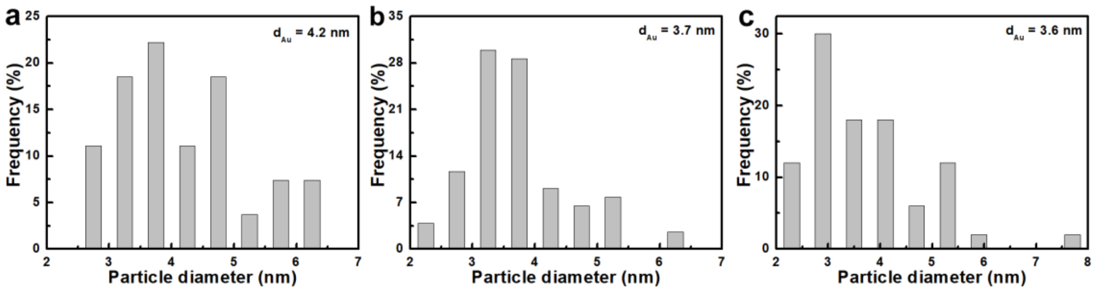

Fig. S2 Size distributions of Au NPs in Au_x/PCN: (**a**) Au_1/PCN, (**b**) $Au_{2.5}$/PCN and (**c**) Au_5/PCN

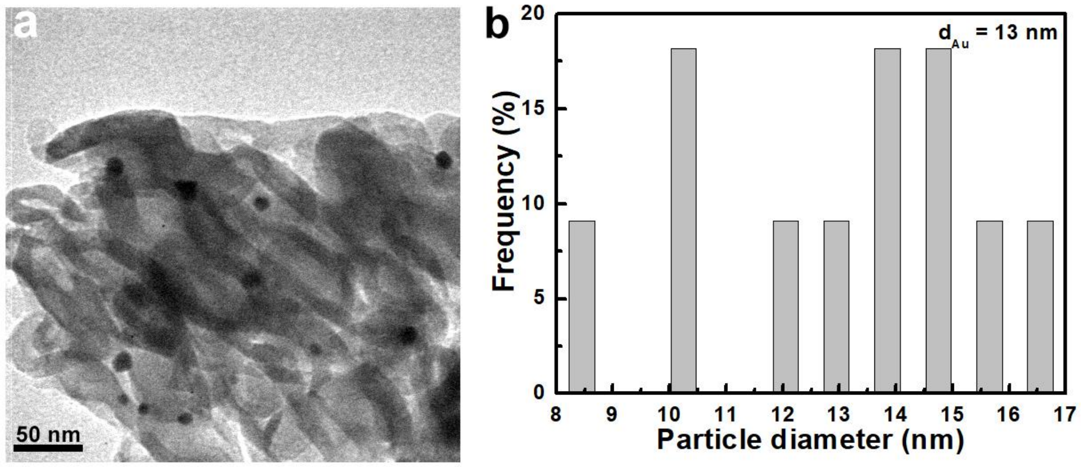

Fig. S3 TEM image of $Au_{2.5}$-PCN (**a**) and the corresponding size distributions of Au NPs in $Au_{2.5}$-PCN (**b**)

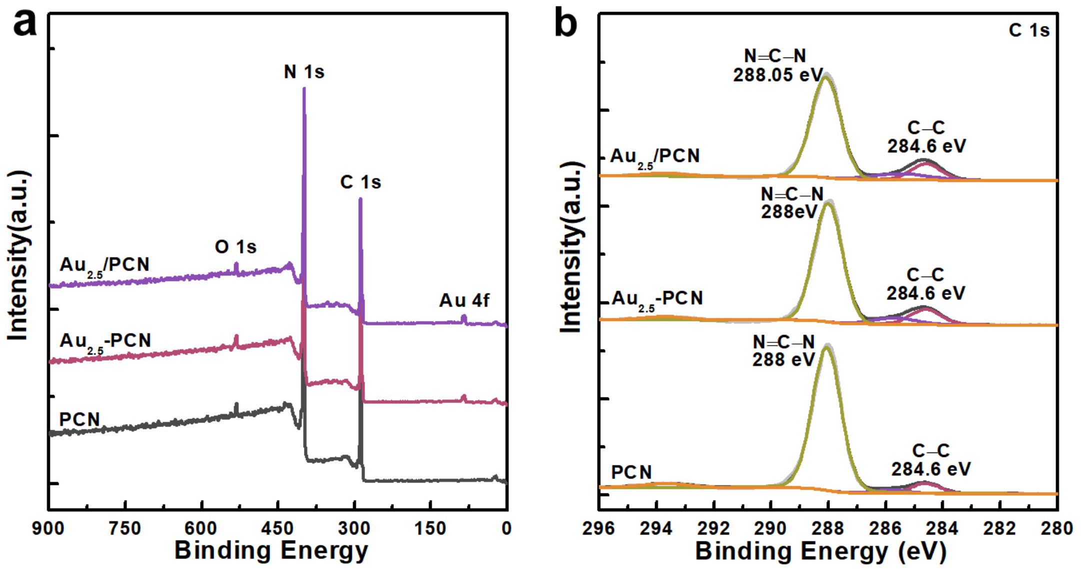

Fig. S4 XPS spectra of $Au_{2.5}$/PCN and reference sample (**a**) survey spectra and (**b**) high-resolution C 1s spectra.

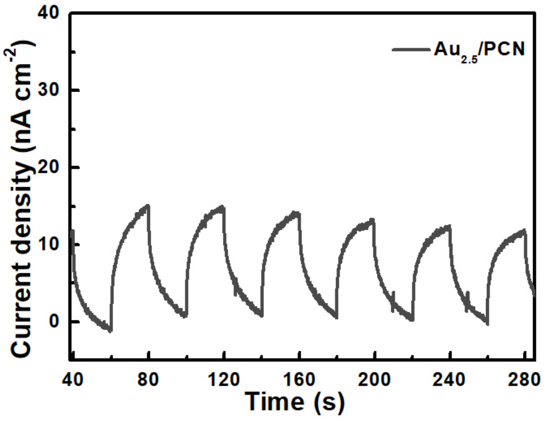

Fig. S5 The transient photocurrent response of $Au_{2.5}$/PCN under irradiation of Xe lamp with 550 nm band-pass filters

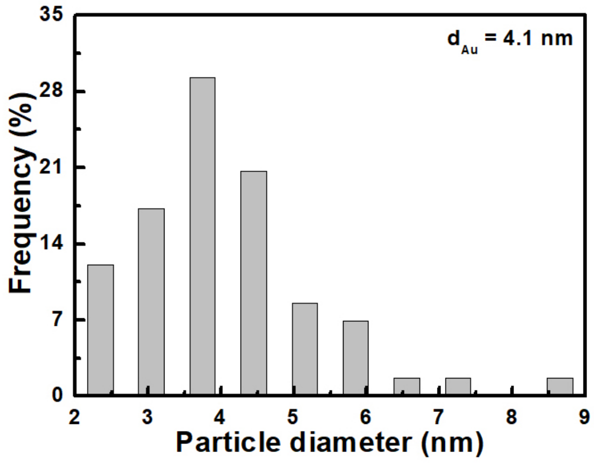

Fig. S6 The size distributions of Au NPs in PtSAs-$Au_{2.5}$/PCN

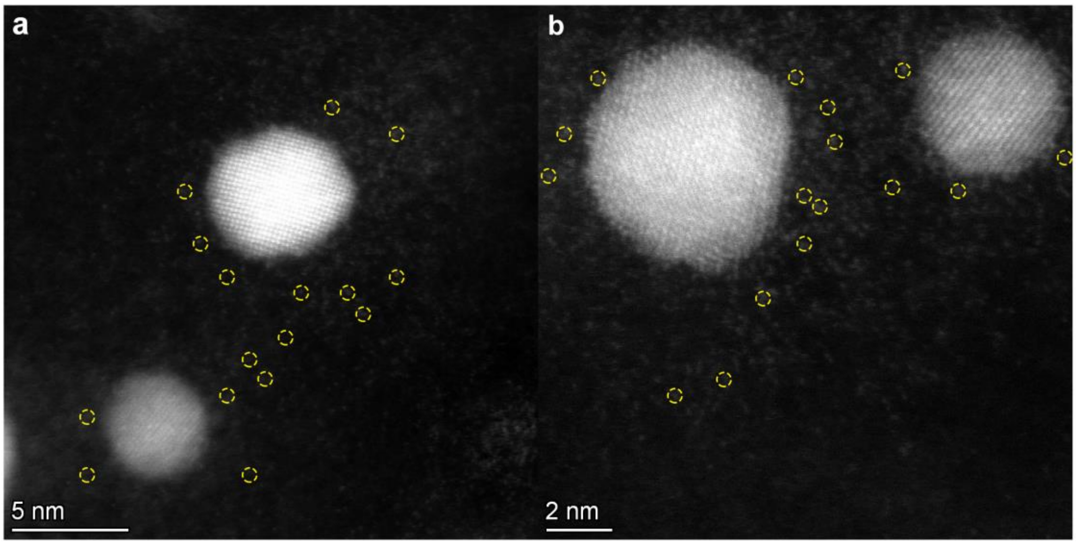

Fig. S7 High-resolution HAADF-STEM images of PtSAs-$Au_{2.5}$/PCN for other regions

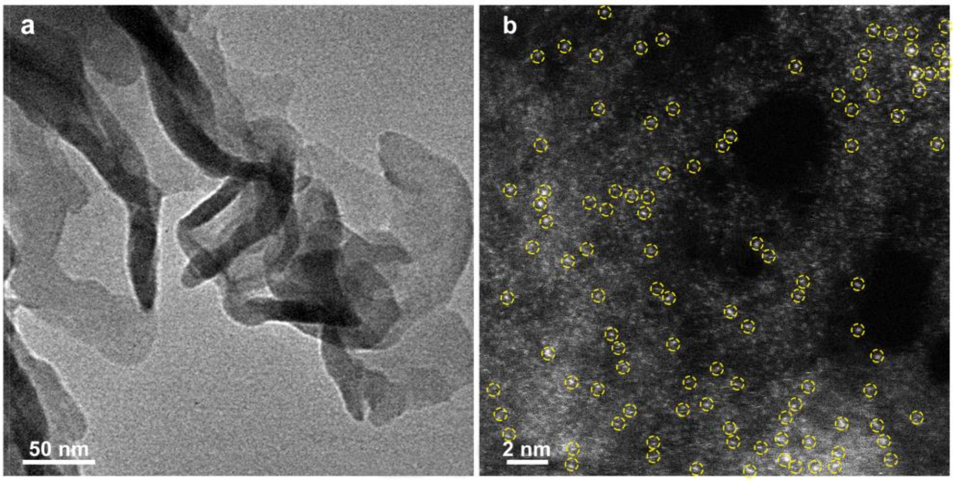

Fig. S8 The TEM (**a**) and high-resolution HAADF-STEM image (**b**) of PtSAs-PCN

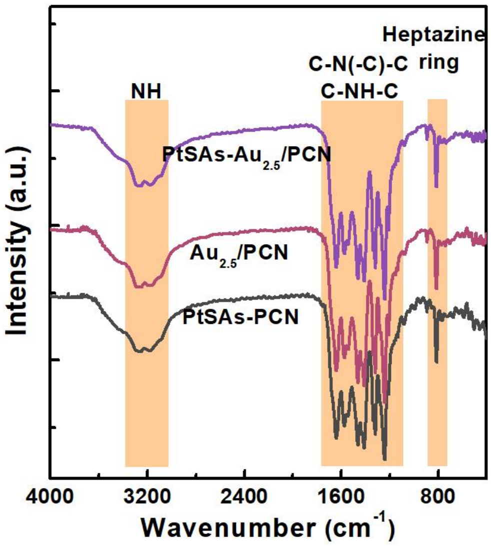

Fig. S9 FTIR spectrums of PtSAs-PCN, $Au_{2.5}$/PCN and PtSAs-$Au_{2.5}$/PCN

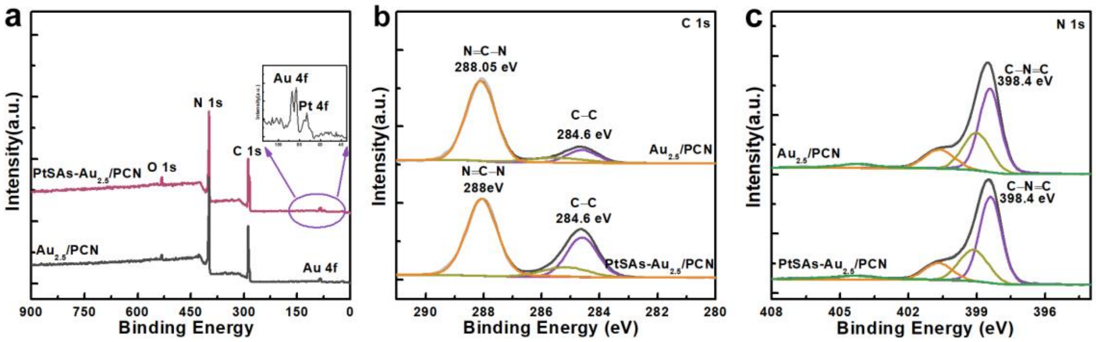

Fig. S10 XPS spectra of PtSAs-$Au_{2.5}$/PCN and reference sample (**a**) survey spectra, (**b**) high-resolution C 1s spectra and (**c**) high-resolution N 1s spectra

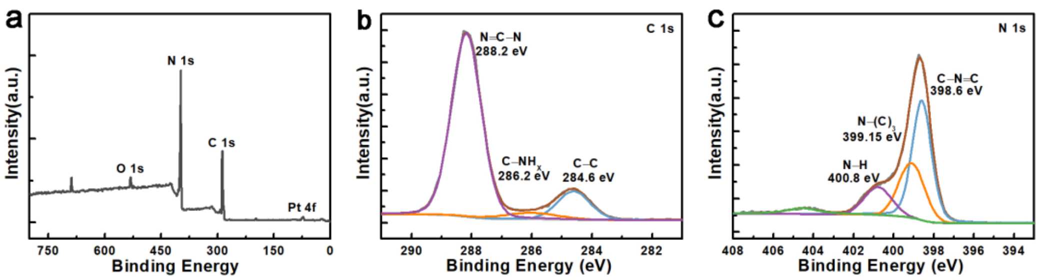

Fig. S11 XPS spectra of PtSAs-PCN and reference sample (**a**) survey spectra, (**b**) high-resolution C 1s spectra and (**c**) high-resolution N 1s spectra

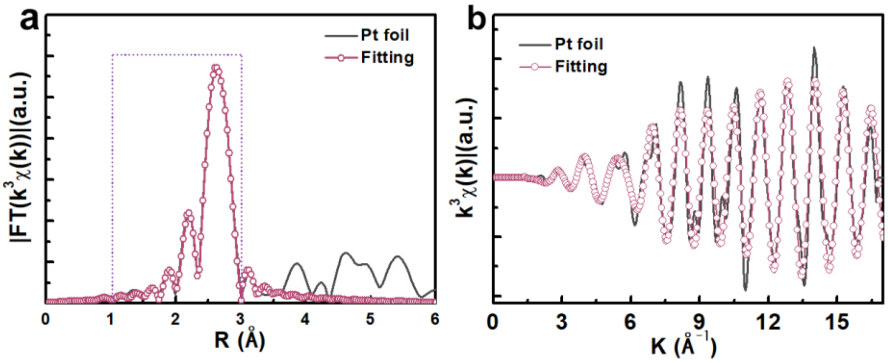

Fig. S12 Pt EXAFS fitting curves of Pt foil at: (**a**) R space and (**b**) K space

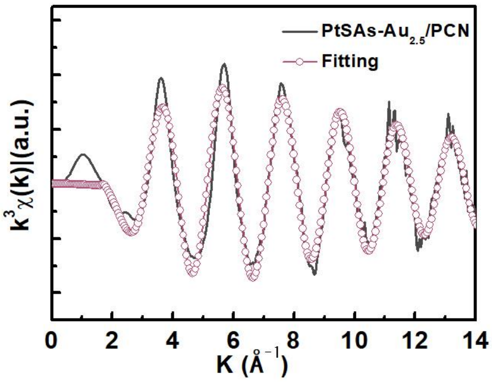

Fig. S13 Pt EXAFS fitting curves of PtSAs-$Au_{2.5}$/PCN at K space

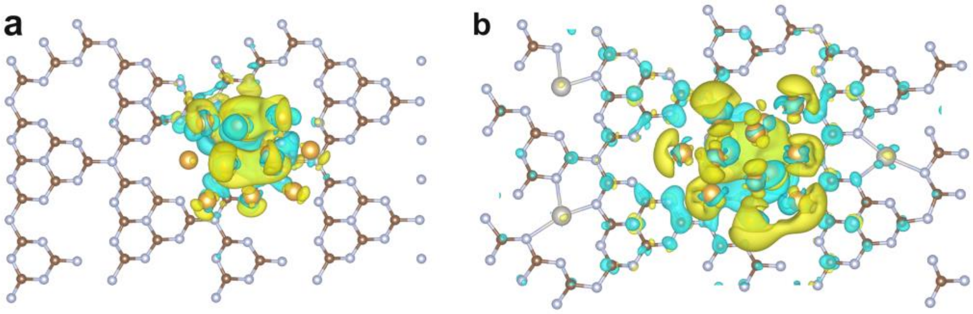

Fig. S14 Difference charge density analysis of (**a**) Au/PCN and (**b**) PtSAs-Au/PCN, yellow and cyan represent electron accumulation and depletion, respectively

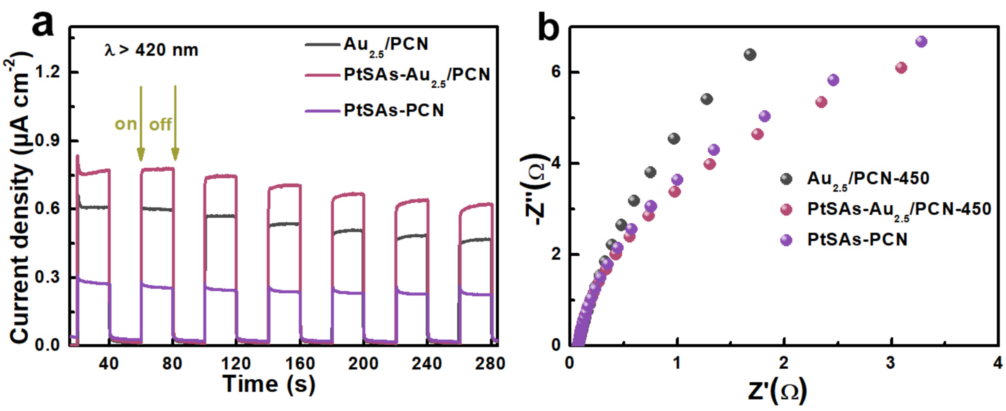

Fig. S15 TPR density (b) and EIS Nyquist plots (c) of PtSAs-$Au_{2.5}$/PCN and reference samples under visible light

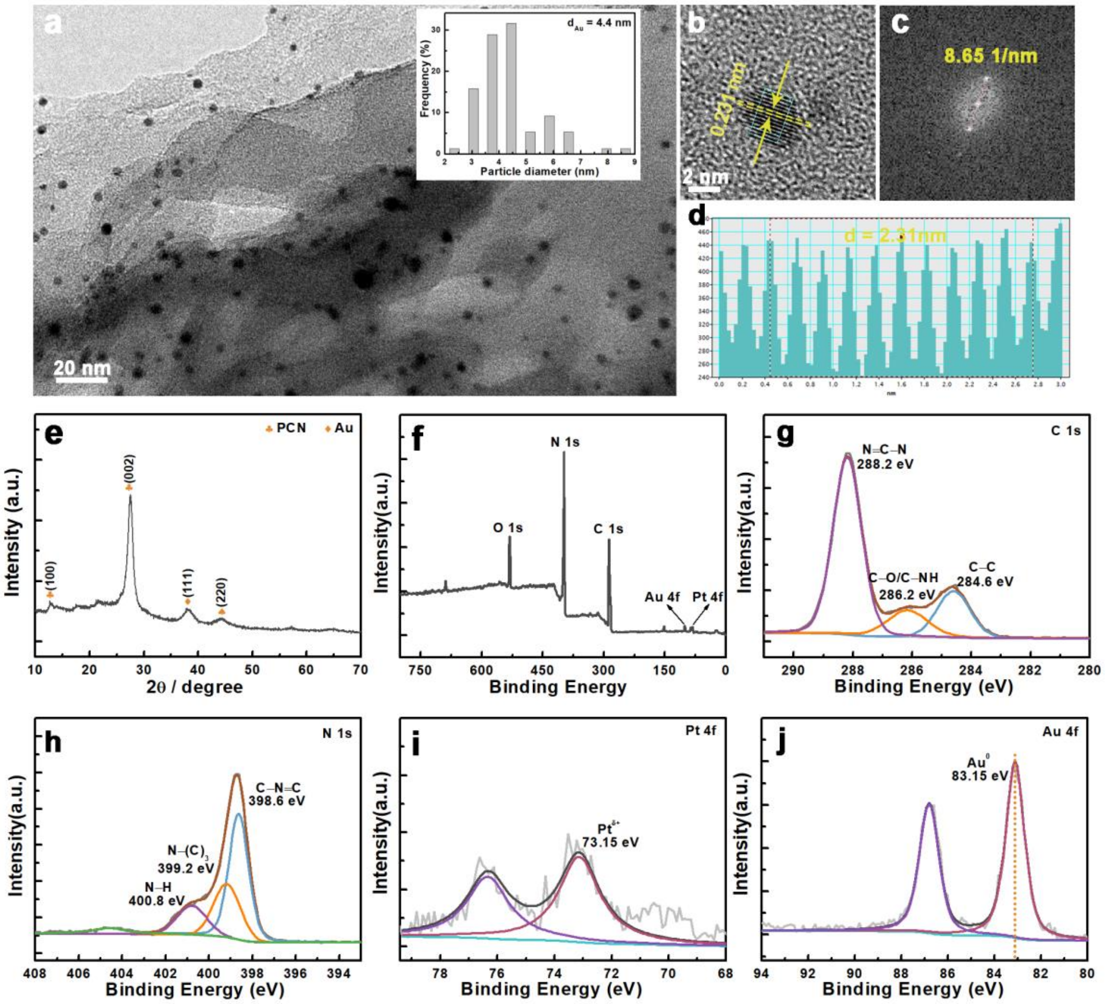

Fig. S16 TEM (**a**) and size distribution (insertion) as well as High-resolution TEM (**b**) image of PtSAs-$Au_{2.5}$/PCN after catalytic test. The FFT (**c**) and Au intensity profile (**d**) of Au NPs in b. (**e**) XRD pattern of PtSAs-$Au_{2.5}$/PCN after catalytic test. (**f-j**) XPS spectra of PtSAs-$Au_{2.5}$/PCN after catalytic test: (**f**) survey spectra, (**g**) C 1s, (H) N 1s, (**i**) Pt 4f and (**j**) Au 4f

Table S1 EXAFS fitting results for PtSAs-$Au_{2.5}$/PCN

Samples	Path	Coordination number	ΔE (eV)	R (Å)	ΔR (Å)	σ^2 (10^{-3} $Å^2$)	R-factor
Pt foil	Pt-Pt	12	7.65	2.76	-0.009	0.005	0.0038
PtSAs-$Au_{2.5}$/PCN	Pt-N	6.09	11.076	1.99	-0.008	0.003	0.004

The so^2 is determined to be 0.837 via the fitting of Pt foil.

Table S2 Summary of previous Au-based PCN plasmonic materials for photocatalytic H_2 evolution activity

Catalysts	Light Source	H_2 evolution rate (mmol g^{-1} h^{-1})	Refs.
Au cluster-NP/C_3N_4 (30 mg)	300 W Xe lamp (λ ≥ 420 nm)	0.23	S1
Au NRs/g-C_3N_4 (100 mg)	300 W Xe lamp (λ ≥ 420 nm)	0.35	S2
Au/g-C_3N_4 (20 mg)	300 W Xe lamp (λ ≥ 420 nm)	0.54	S3
Au/SnO_2/g-C_3N_4 (100 mg)	300 W Xe lamp (λ ≥ 400 nm)	0.77	S4
PtAu/g-C_3N_4 (50 mg)	300 W Xe lamp	1.01	S5
Au/g-C_3N_4-AAPC (20 mg)	150 W Xe lamp (Solar Light)	About 1.3	S6
TiO_2-BCN-AuCu (20 mg)	300 W Xe lamp	2.15	S7
Au/g-C_3N_4 (4 hours) (100 mg)	250 W halide lamp (λ ≥ 380 nm)	2.3	S8
$W_{18}O_{49}$/Au/g-C_3N_4 (20 mg)	300 W Xe lamp (1 sun irradiation)	3.46	S9
g-C_3N_4/Fe_2O_3/Pt/Au (50 mg)	150 W Xe lamp (Solar Light)	4.73	S10
Pt@Au NR_{769}/$CNNT_{650}$ (20 mg)	300 W Xe lamp (λ ≥ 420 nm)	10.35	S11
Pt-CN (50 mg)	300 W Xe lamp	6.36	S12
PtSA-CN620 (20 mg)	300 W Xe lamp (λ ≥ 420 nm)	3.02	S13
PtSAs/C_3N_4	300 W Xe lamp	11.47	S14
Pt-SA/CN	300 W Xe lamp	1.4	S15
PtSAs-$Au_{2.5}$/PCN (10 mg)	300 W Xe lamp (λ ≥ 420 nm)	13.70	This work

Supplementary References

[S1] R. Cheng, H. Su, F. M. Tang, W. Che, Y. Y. Huang et al., Synergetic enhancement of plasmonic hot-electron injection in au cluster-nanoparticle/C_3N_4 for photocatalytic hydrogen evolution. J. Mater. Chem. A **5**(37), 19649-19655 (2017). https://doi.org/10.1039/C7TA03808A

[S2] H. Y. Tian, X. Liu, Z. Q. Liang, P. Y. Qiu, X. Qian et al., Gold nanorods/g-c_3n_4 heterostructures for plasmon-enhanced photocatalytic H_2 evolution in visible and near-infrared light. J. Colloid Interface Sci. **557**, 700-708 (2019). https://doi.org/10.1016/j.jcis.2019.09.075

[S3] Y. Z. Guo, H. L. Jia, J. H. Yang, H. Yin, Z. Yang et al., Understanding the roles of plasmonic Au nanocrystal size, shape, aspect ratio and loading amount in Au/g-C_3N_4 hybrid nanostructures for photocatalytic hydrogen generation. Phys Chem Chem. Phys. **20**(34), 22296-22307 (2018). https://doi.org/10.1039/C8CP04241A

[S4] A. Zada, M. Humayun, F. Raziq, X. L. Zhang, Y. Qu et al., Exceptional visible-light-driven cocatalyst-free photocatalytic activity of g-C_3N_4 by well designed nanocomposites with plasmonic au and SnO_2. Adv. Energy Mater. **6**(21), 1601190 (2016). https://doi.org/10.1002/aenm.201601190

[S5] K. Bhunia, M. Chandra, S. Khilari, D. Pradhan, Bimetallic ptau alloy nanoparticles-integrated g-C_3N_4 hybrid as an efficient photocatalyst for water-to-hydrogen conversion. ACS Appl. Mater. Interfaces **11**(1), 478-488 (2019). https://doi.org/10.1021/acsami.8b12183

[S6] C.-C. Nguyen, M. Sakar, M.-H. Vu, T.-O. Do, Nitrogen vacancies-assisted enhanced plasmonic photoactivities of Au/g-C_3N_4 crumpled nanolayers: A novel pathway toward efficient solar light-driven photocatalysts. Ind. Eng. Chem. Res. **58**(9), 3698-3706 (2019). https://doi.org/10.1021/acs.iecr.8b05792

[S7] P. Babu, S. R. Dash, K. Parida, Mechanistic insight the visible light driven hydrogen generation by plasmonic Au-Cu alloy mounted onTiO_2 @b-doped g-C_3N_4 heterojunction photocatalyst. J. Alloys Compd. **909**, 164754 (2022). https://doi.org/10.1016/j.jallcom.2022.164754

[S8] M. Caux, H. Menard, Y. M. AlSalik, J. T. S. Irvine, H. Idriss, Photo-catalytic hydrogen production over au/g-c_3n_4: Effect of gold particle dispersion and morphology. Phys. Chem. Chem. Phys. **21**(29), 15974-15987 (2019). https://doi.org/10.1039/C9CP02241D

[S9] I. Hong, Y.-A. Chen, Y.-J. Hsu, K. Yong, Triple-channel charge transfer over $W_{18}O_{49}$/Au/g-C_3N_4 Z-scheme photocatalysts for achieving broad-spectrum solar hydrogen production. ACS Appl. Mater. Interfaces. **13**(44), 52670-52680 (2021). https://doi.org/10.1021/acsami.1c15883

[S10] S. A. Hassanzadeh-Tabrizi, C.-C. Nguyen, T.-O. Do, Synthesis of Fe_2O_3/Pt/Au nanocomposite immobilized on g-C_3N_4 for localized plasmon photocatalytic hydrogen evolution. Appl. Surf. Sci. **489**(741-754 (2019). https://doi.org/10.1016/j.apsusc.2019.06.010

[S11] L. S. Zhang, N. Ding, L. C. Lou, K. Iwasaki, H. J. Wu et al., Localized surface plasmon resonance enhanced photocatalytic hydrogen evolution via Pt@Au NRs/ C_3N_4 nanotubes under visible-light irradiation. Adv. Funct. Mater. **29**(3), 1806774 (2019). https://doi.org/10.1002/adfm.201806774

[S12] X. Li, W. Bi, L. Zhang, S. Tao, W. Chu et al., Single-atom Pt as co-catalyst for enhanced photocatalytic H_2 evolution. Adv. Mater. **28**(12), 2427-2431 (2016). https://doi.org/10.1002/adma.201505281

[S13] P. Zhou, F. Lv, N. Li, Y. Zhang, Z. Mu et al., Strengthening reactive metal-support interaction to stabilize high-density pt single atoms on electron-deficient g-C_3N_4 for boosting photocatalytic H_2 production. Nano Energy **56**, 127-137 (2019). https://doi.org/10.1016/j.nanoen.2018.11.033

[S14] Y. Hu, Y. Qu, Y. Zhou, Z. Wang, H. Wang et al., Single Pt atom-anchored C_3N_4: A bridging Pt–N bond boosted electron transfer for highly efficient photocatalytic h_2 generation. Chem. Eng. J. **412**, 128749 (2021). https://doi.org/10.1016/j.cej.2021.128749

[S15] Y. Cao, D. Wang, Y. Lin, W. Liu, L. Cao et al., Single Pt atom with highly vacant d-orbital for accelerating photocatalytic H_2 evolution. ACS Appl. Energy Mater. **1**(11), 6082-6088 (2018). https://doi.org/10.1021/acsaem.8b01143
